# The allosteric modulation of lipases and its possible biological relevance

**DOI:** 10.1186/1742-4682-4-34

**Published:** 2007-09-07

**Authors:** Jens Köhler, Bernhard Wünsch

**Affiliations:** 1 Institut für Pharmazeutische und Medizinische Chemie, Westfälische Wilhelms-Universität Münster, Hittorfstraße 58-62, D-48149 Münster, Germany

## Abstract

**Background:**

During the development of an enantioselective synthesis using the lipase from *Mucor miehei *an unusual reaction course was observed, which was analyzed precisely. For the first time an allosteric modulation of a lipase changing its selectivity was shown.

**Theory:**

Considering the biological relevance of the discovered regulation mechanism we developed a theory that describes the regulation of energy homeostasis and fat metabolism.

**Conclusion:**

This theory represents a new approach to explain the cause of the metabolic syndrome and provides an innovative basis for further research activity.

## Background

### Introduction

Asymmetric syntheses are investigated to produce chiral organic compounds with high enantiomeric purity. Their development has been an expending task of research during the last years. Valuable tools to perform the required chemical reactions are enzymes, which work as catalysts. The substrate to be transformed binds to the chiral binding site of the employed enzyme and is modified enantioselectively. Very often lipases are used for this kind of transformation [1,2]. In water these enzymes catalyze the hydrolysis of esters to afford alcohols and acids. This reaction corresponds to their natural task, hydrolysis of triglycerides. Their catalytic activity is increased by interfacial activation [3]. Since lipases are also stable and active in neat organic solvents, their use as catalysts is very convenient. In organic solvents the equilibrium of the catalyzed reaction is shifted to the direction of esters, which are formed instead of hydrolyzed. Often transesterfications are carried out to produce esters from alcohols. The most useful acyl donors for this feature are enol esters, e.g. vinyl or isopropenyl acetate, as the resulting enols tautomerize into carbonyl compounds. This procedure makes the reaction almost irreversible [4].

In our experiments two different lipases were used. We employed the lipase from *Burkholderia cepacia *and the lipase from *Mucor miehei*, which are common in organic synthesis. In numerous publications both lipases were investigated and described in detail, their tertiary structures have been characterized by X-ray structure analysis [2-7]. Due to reclassification *Burkholderia cepacia *was renamed during the last years. Therefore the lipase originating from this bacterium can also be labeled as lipase from *Pseudomonas species*, *Pseudomonas cepacia *or *Pseudomonas fluoreszens*. The preparation we used in our experiments is commercially available as Amano lipase PS-CII, which is the lipase from *B. cepacia *immobilized on ceramic particles. The immobilized enzyme forms better suspensions in organic solvents, has an increased activity, can be recycled by filtration and is therefore more convenient to use.

The lipase from *Mucor miehei *is also found as lipase from *Rhizomucor miehei*. Both its amino acid sequence and tertiary structure are known [8-10]. Like almost all other lipases the lipase from *M. miehei *also shows interfacial activation due to a lid at its active site, which can switch between a closed and open form. The genetic information of this lipase was inserted into *Aspergillus oryzae *[11]. Thus, the lipase from *Mucor miehei *is inexpensively available in pure form and great amounts using this expression vector [12]. The preparation, which was used in our experiments, was produced by this procedure and immobilized on an ion-exchange resin. It is commercially available from Novo Nordisk as Lipozyme^®^.

### Findings

**Figure 1:**
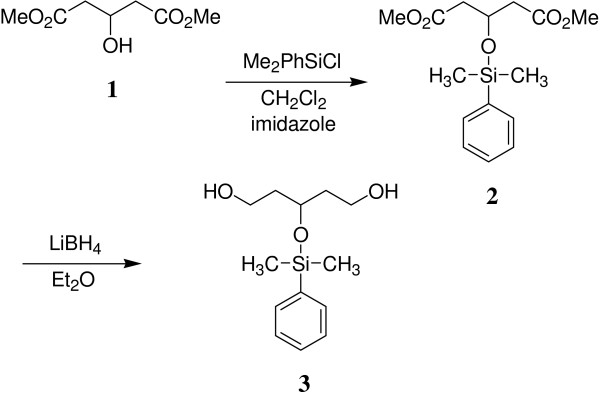
Synthesis of the prochiral diol **3**.

Herein, we describe the asymmetric transformation of a prochiral diol with the lipases from *Burkholderia cepacia *and *Mucor miehei*. The detailed reaction conditions of the experiments, the spectroscopic and analytical data of all products and the employed procedures were published recently [13]. The relevant part of the work is summarized as follows:

The synthesis was started from prochiral diester **1**, which was silylated with chloro-dimethyl-phenyl-silane. The resulting silyl ether **2 **was reduced to give the prochiral pentanediol **3 **(Fig. 1).

**Figure 2:**
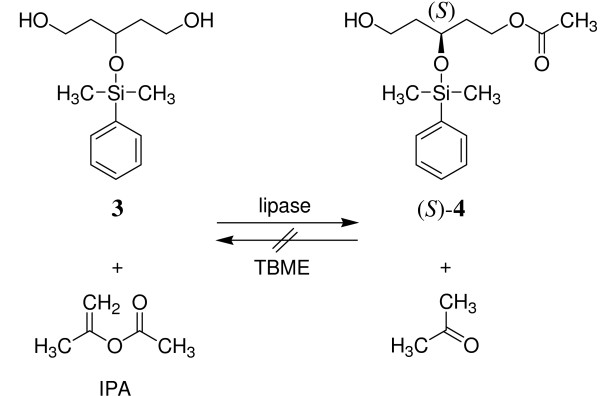
Lipase catalyzed enantioselective, irreversible acetylation.

The lipase should acetylate the prochiral diol **3 **enantioselectively by irreversible transesterification with isopropenyl acetate (IPA) using *tert*-butyl methyl ether (TBME) as solvent to provide enantiopure monoacetate (*S*)-**4 **(Fig. 2).

**Figure 3:**
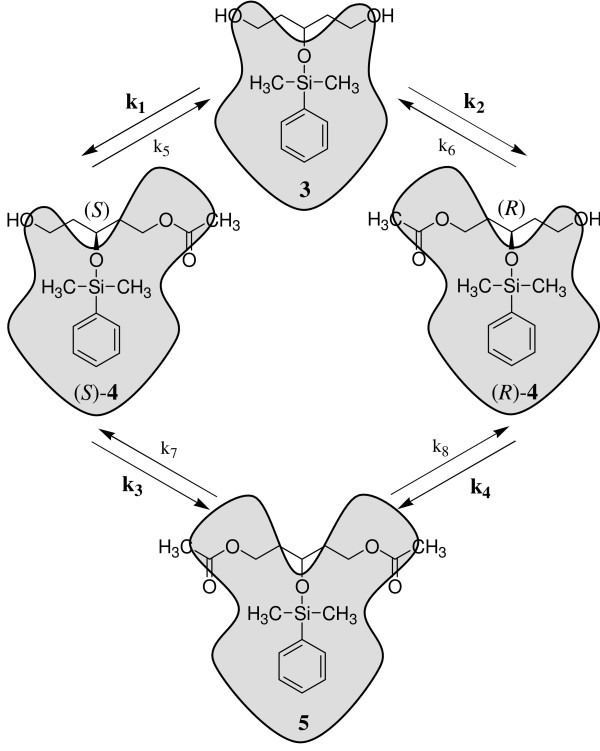
Lipase catalyzed acetylation of the prochiral diol **3**.

However, acetylation of the prochiral diol **3 **can take place at either of the OH groups to yield the enantiomeric monoacetates (*S*)-**4 **and (*R*)-**4 **or at both OH groups to provide the prochiral diacetate **5 **(Fig. 3). In order to illustrate this reaction sequence, contour plots representing the structural features of the respective chemical compounds are introduced in Figure 3.

The lipase catalyzed acetylation of diol **3 **was monitored by HPLC analysis of samples taken from the reaction mixture using an achiral RP-18 column as well as a chiral column. In this way the development of the amounts of substances **3**, **4 **and **5 **and the development of the enantiomeric excess of monoacetate (*S*)-**4 **were recorded and displayed as reaction courses. The absolute configuration of monoacetate (*S*)-**4 **was determined by CD spectroscopy [13].

**Figure 4:**
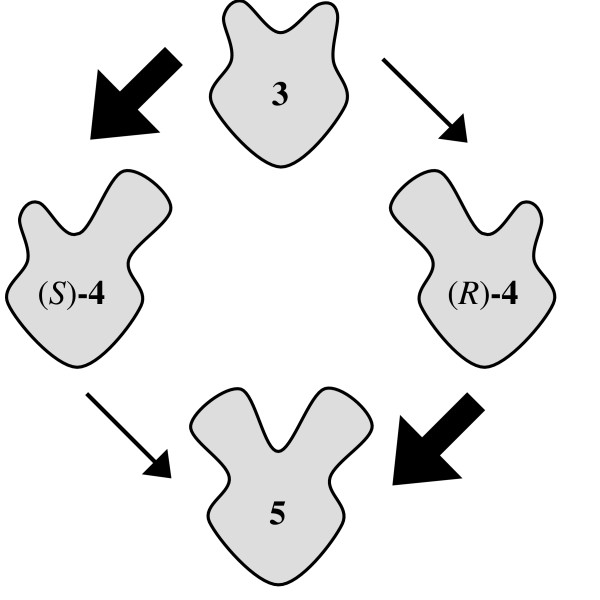
Enantioselectivity of both lipases (lipase from *B. cepacia *and from *M. miehei*).

In the first step of the reaction both lipases catalyzed the acetylation of diol **3 **yielding preferentially the (*S*)-configured monoacetate (*S*)-**4**. The enantiomeric excess increased during the progress of the reaction, because the small amount of formed (*R*)-configured monoacetate (*R*)-**4 **containing the preferred free OH-group was acetylated faster than (*S*)-**4 **to provide the prochiral diacetate **5 **in the second step of the reaction (Figure 4).

**Figure 5:**
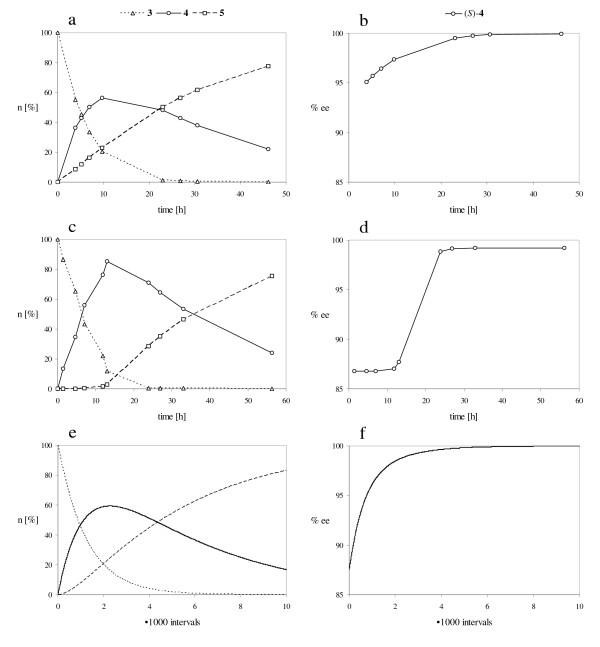
Progress of the reaction carried out at +20°C; a, c, e: Amount of compounds **3**, **4 **and **5 **(n [%]); b, d, f: Enantiomeric excess of (*S*)-**4 **(% ee); a, b: Transformation catalyzed by lipase from *B. cepacia*; c, d: Transformation catalyzed by lipase from *M. miehei*; e, f: Simulation of the reaction using a constant lipase activity *a *= 0.004 [14]; The rate constants *k*_1 _to *k*_8 _are defined in Figure 3; *k*_1 _= 15, *k*_2 _= 1, *k*_3 _= 4, *k*_4 _= 60, *k*_5 _= 15·10^-6^, *k*_6 _= 1·10^-6^, *k*_7 _= 4·10^-6^, *k*_8 _= 60·10^-6^.

However, the reaction courses produced by the lipases from *Burkholderia cepacia *and *Mucor miehei *differed in a very interesting manner (Fig. 5a, b and Fig. 5c, d). The reaction catalyzed by the lipase from *M. miehei *led to a higher concentration of monoacetate **4 **during the progress of the reaction (Fig. 5c) than the lipase from *B. cepacia *(Fig. 5a). This is an amazing result, since the enantiomeric excess of (*S*)-**4 **produced by the lipase from *B. cepacia *(Fig. 5b) was higher than the one produced by the lipase from *M. miehei *(Fig. 5d). Obviously diol **3 **was acetylated selectively by the lipase from *M. miehei *to give the monoacetates (*S*)-**4 **and (*R*)-**4 **in the first step of the reaction. The second acetylation of both monoacetates (*S*)-**4 **and (*R*)-**4 **did not take place until diol **3 **was consumed almost completely. The explanation for this unusual observation is discussed in the following parts of the manuscript.

### Computer simulation

Helpful for the interpretation of the results described above is a computer simulation of the processes using a mathematical model of the reaction scheme shown in Figure 3. According to this mathematical model the reaction course is divided into several small time intervals within the reaction conditions can assumed to be constant [14]. The progress of the reaction is simulated by subdividing the activity of the catalyst according to the competing reactions. It is considered that the compounds react according to their current concentration, their respective affinity towards the lipase and the rate constant of the proceeding partial reaction. This model was programmed as a Microsoft^® ^Excel spreadsheet and used to simulate the investigated asymmetric transformations.

The prochiral diol **3 **is converted into the (*S*)- or (*R*)-configured monoacetates (*S*)-**4 **and (*R*)-**4 **with different reaction rate constants *k*_1 _and *k*_2 _in the first reaction step. In the second step the enantiomeric monoacetates (*S*)-**4 **and (*R*)-**4 **are converted enantioselectively with different reaction rate constants *k*_3 _or *k*_4 _to give the prochiral diacetate **5 **(Figure 3). Thus, the four reaction rate constants *k*_1 _to *k*_4 _define the four possible acetylation reactions. As lipases catalyze reaction equilibria, the corresponding backward reactions defined by the reaction rate constants *k*_5 _to *k*_8 _can also take place, theoretically. As outlined above the backward reactions are prevented almost completely by employing an enolester for transesterfication. Since the forward reactions are almost irreversible, the corresponding reaction equilibria are set to 10^6^:1 on product side for all simulations carried out (*k*_1_/*k*_5 _= *k*_2_/*k*_6 _= *k*_3_/*k*_7 _= *k*_4_/*k*_8 _= 10^6^). In addition the activity of the lipase is defined by the variable *a*. Thus, the properties of a given lipase can be defined by setting values for its activity *a *and the reaction rate constants *k*_1 _to *k*_8_.

Figure 5 (e, f) shows the simulated progress of a reaction using a lipase with an enantioselectivity of 15:1 for both acetylation steps (*k*_1_/*k*_2 _= *k*_4_/*k*_3 _= *k*_5_/*k*_6 _= *k*_8_/*k*_7 _= 15). It is assumed that the second acetylation takes place four times faster than the first one (*k*_4_/*k*_1 _= *k*_3_/*k*_2 _= *k*_8_/*k*_5 _= *k*_7_/*k*_6 _= 4) and that the activity of the lipase remains constant (*a *= 0.004).

### Allosteric effect

**Figure 6:**
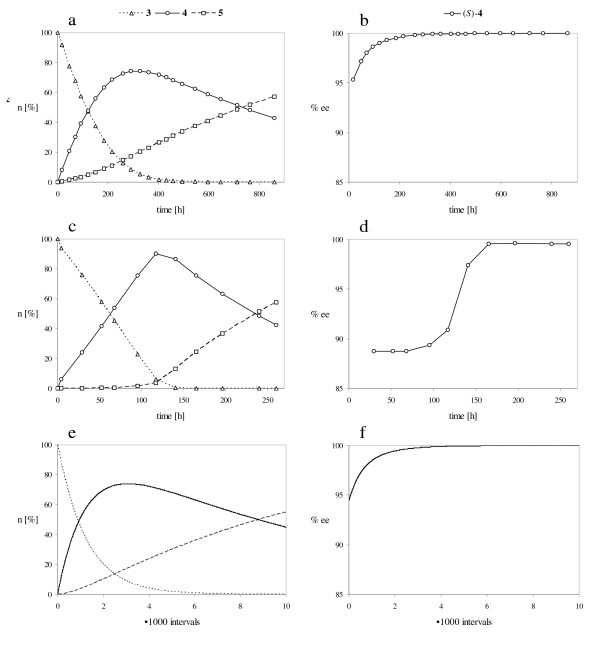
Progress of the reaction carried out at low temperature; a, c, e: Amount of compounds **3**, **4 **and **5 **(n [%]); b, d, f: Enantiomeric excess of (*S*)-**4 **(% ee); a, b: Transformation catalyzed by lipase from *B. cepacia *at -40°C; c, d: Transformation catalyzed by lipase from *M. miehei *at -10°C; e, f: Simulation of the reaction using a constant lipase activity *a *= 0.004 [14]; The rate constants *k*_1 _to *k*_8 _are defined in Figure 3; *k*_1 _= 35, *k*_2 _= 1, *k*_3 _= 4, *k*_4 _= 140, *k*_5 _= 35·10^-6^, *k*_6 _= 1·10^-6^, *k*_7 _= 4·10^-6^, *k*_8 _= 140·10^-6^.

The comparison of the experimentally determined reaction courses with the simulated ones leads to the following results. The reaction performed with lipase from *B. cepacia *corresponds to the prediction made by the simulation (Fig. 5a, b and Fig. 5e, f). In further experiments it was shown that the enantioselectivity of the lipase was increased by lowering the reaction temperature, whereas the selectivity to perform either the first or second acetylation step remained constant (Fig. 5a, b and Fig. 6a, b) [13].

In contrast to the *B. cepacia *lipase catalyzed transformation the reaction course produced by the lipase from *M. miehei *does not correspond to the simulation, provided that the activity of the lipase remains constant during the reaction time. It is remarkable that diacetate **5 **was not produced at the beginning of the reaction and the enantiomeric excess remained constant during this time period. In order to verify the differing catalytic properties of the two lipases the experiments were repeated at low temperature. Figure 6 shows the progress of the reactions carried out with the lipases from *B. cepacia *at -40°C (a, b) and *M. miehei *at -10°C (c, d).

**Figure 7:**
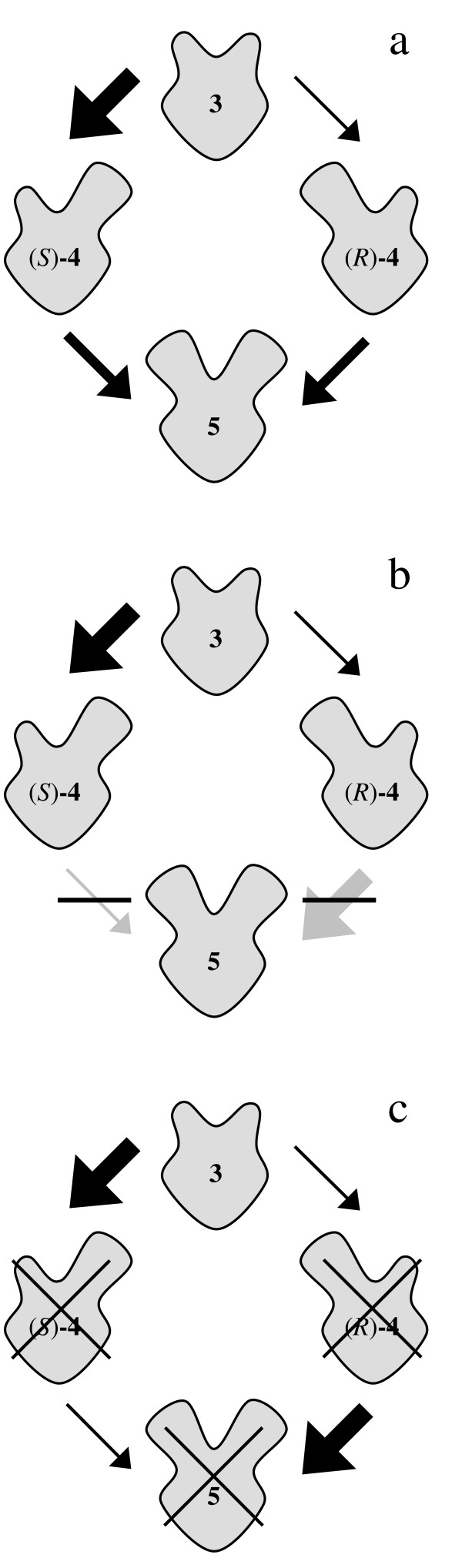
Possible explanations for a constant enantiomeric excess during the progress of the reaction; a: non-enantioselective transformation of (*S*)-**4 **and (*R*)-**4 **into **5**; b: inhibited transformation of (*S*)-**4 **and (*R*)-**4 **into **5**; c: non-competitive, since compounds **4 **and **5 **do not bind to the enzyme (indicated by crosses).

Once again the experimentally determined reaction course produced by the lipase from *B. cepacia *is in good agreement with the computer simulation (Fig. 6a, b and Fig. 6e, f). On the contrary, the enantiomeric excess remained constant again at the beginning of the reaction using the lipase from *M. miehei *(Fig. 6d, compare Fig. 5d). Due to the reaction kinetics a constant enantiomeric excess is only possible, if the second acetylation of monoacetates (*S*)-**4 **and (*R*)-**4 **into diacetate **5 **proceeds non-enantioselectively (*k*_3 _= *k*_4 _and *k*_7 _= *k*_8_) (Fig. 7a) or does not take place (*k*_3 _= *k*_4 _= 0_i→∞_) (Fig. 7b).

**Figure 8:**
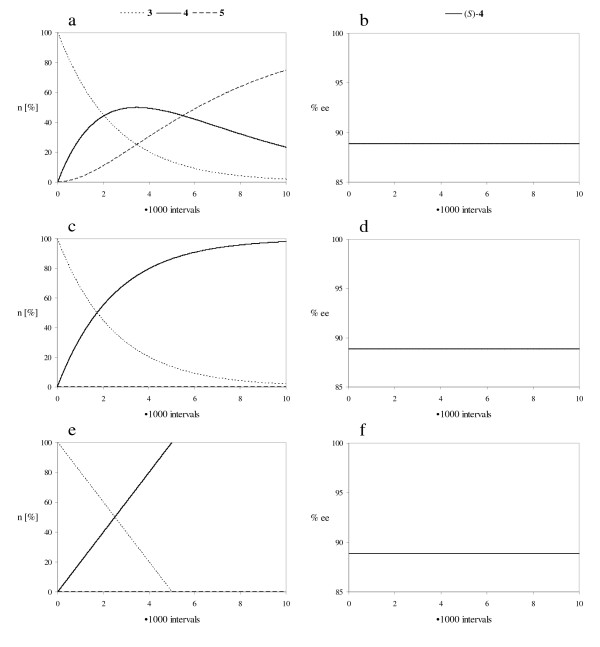
Simulated reaction courses using a constant lipase activity *a *[14]; The rate constants *k*_1 _to *k*_8 _are defined in Figure 3; a, c, e: Amount of compounds **3**, **4 **and **5 **(n [%]); b, d, f: Enantiomeric excess of (*S*)-**4 **(% ee); a, b: non-enantioselective transformation of (*S*)-**4 **and (*R*)-**4 **into **5 **(*a *= 0.0008); *k*_1 _= 17, *k*_2 _= 1, *k*_3 _= 9, *k*_4 _= 9, *k*_5 _= 17·10^-6^, *k*_6 _= 1·10^-6^, *k*_7 _= 9·10^-6^, *k*_8 _= 9·10^-6^; c, d: inhibited transformation of (*S*)-**4 **and (*R*)-**4 **into **5 **(*a *= 0.0004); *k*_1 _= 17, *k*_2 _= 1, *k*_3 _= 0.009, *k*_4 _= 0.009, *k*_5 _= 17·10^-6^, *k*_6 _= 1·10^-6^, *k*_7 _= 0.009·10^-6^, *k*_8 _= 0.009·10^-6^; e, f: non-competitive, since compounds **4 **and **5 **do not bind to the enzyme (*a *= 0.02); *k*_1 _= 17, *k*_2 _= 1.

In Figure 8 the progress of the reactions is simulated for these supposed situations. A non-enantioselective second acetylation of monoacetates (*S*)-**4 **and (*R*)-**4 **would result in a rapid formation of diacetate **5 **(Fig. 8a), which is not seen in the experiment (Fig. 6c). According to the remaining second possibility, the acetylation of monoacetates (*S*)-**4 **and (*R*)-**4 **does not take place at the beginning of the reaction (Fig. 8c, d). Obviously, an inhibition occurs, which is reversed during the reaction.

However, this simulation does still not match the experimental data exactly (Fig. 6c, d), since both the decrease of diol **3 **and the increase of monoacetate **4 **proceeded linearly in the experiment. This indicates that the transformation does not depend on the concentration of compounds **3 **and **4**. Substrates **3**, **4 **and **5 **do not compete with each other, since only diol **3 **can bind to the active site of the lipase (Fig. 7c). However, the computer simulation used up to now is mathematically based on a competition situation and has to be modified to take the non-competitive situation into account. Differing from literature [14] the changes of the amounts of substances during a time interval are then as follows:

Δn(**3**)_i _= - *a*

Δn((*S*)-**4**)_i _= *a*·*d*

Δn((*R*)-**4**)_i _= *a*·*e*

Δn(**5**)_i _= 0

**Figure 9:**
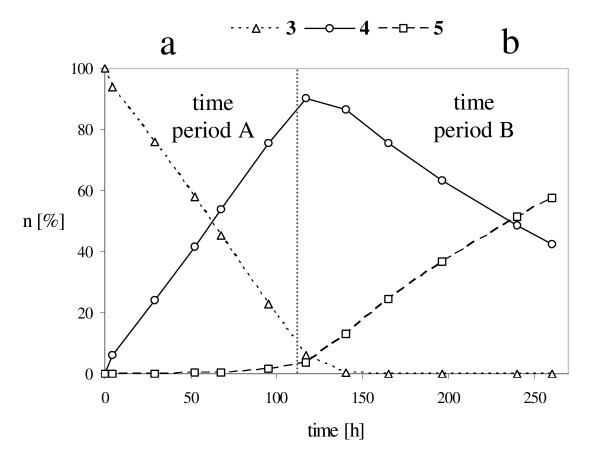
Progress of the reaction carried out at -10°C using lipase from *M. miehei*; Amount of compounds **3**, **4 **and **5 **(n [%]); The reaction course is divided into two time periods: a: period A (compounds **4 **and **5 **do not bind to the lipase); b: time period B (the reaction is competitive).

Using these settings the simulation results in the same linear development of the amounts of diol **3 **and monoacetate **4 **as observed in the experiment at the beginning of the reaction (Fig. 8e, compare Fig. 6c). Obviously the lipase is modulated in a way that only diol **3 **interacts with the active binding site during this time period. Monoacetate **4 **and diacetate **5 **do not compete for the active site. Hence, the reaction is subdivided into two time periods with a different selectivity of the lipase (Fig. 9).

**Figure 10:**
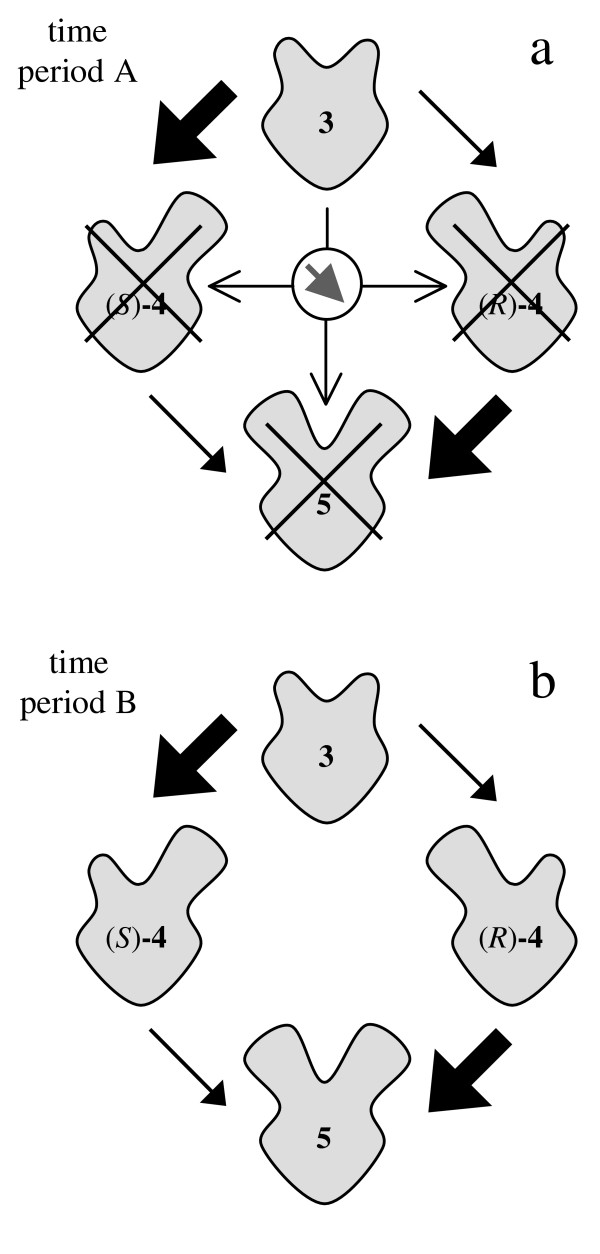
Reaction schemes that explain the progress of the reaction using lipase from *M. miehei *(Fig. 9); a: during time period A compounds **4 **and **5 **do not bind to the lipase (indicated by crosses).; b: during time period B the reaction is competitive.

During time period A (Fig. 9a) the transformation of monoacetate **4 **into diacetate **5 **is inhibited and does not take place, as these compounds cannot bind (Fig. 10a). During time period B (Fig. 9b) this inhibition is reversed and monoacetate **4 **is acetylated (Fig. 10b).

According to this observation the lipase from *M. miehei *is modulated reversibly and inhibited selectively. However, the question remains, whereby this modulation is induced and reversed subsequently. The reason must be a compound, which decreases in concentration during time period A, so that the modulation is reversed at the beginning of time period B due to its very low concentration. This property is only given for diol **3**. An activation of the lipase caused by a compound increasing in concentration is not possible, since this would lead to an increasing activity of the lipase and therefore a non-linear change of the amounts of compounds **3 **and **4**. Hence, diol **3 **used as a reactant must be the reason for the modulation of the lipase. During time period A the transformation of monoacetate **4 **is inhibited by diol **3**. Monoacetate **4 **is acetylated not until diol **3 **is consumed almost quantitatively.

Inhibition of enzymes can be induced by inhibitors binding at the active site or allosterically. The former possibility is always competitive, because the inhibitor competes with the substrate for the active site of the enzyme. A competitive inhibition would lead to altering reaction rates depending on the concentrations of compounds **3 **and **4**. However, a change of the reaction rates was not observed in our experiments. Independent on the concentrations the reaction rates were constant during time period A. Therefore the observed inhibition has to be induced allosterically. In this case the non-competitive and very rare uncompetitive mechanism must be differentiated [15]. According to an uncompetitive mechanism diol **3 **would bind to the substrate-lipase-complex inhibiting its transformation into diacetate-lipase-complex, but not inhibiting its transformation into monoacetate-lipase-complex. In this situation, the diol-lipase-complex and the monoacetate-lipase-complex would compete for the remaining diol **3 **and the reaction rates during time period A would alter depending on the concentrations of compounds **3 **and **4**. Since the experimental data do not display a change of the reaction rates, only a non-competitive mechanism remains possible. Diol **3 **binds allosterically whether the lipase is complexed or not. Therefore, we conclude that the lipase from *M. miehei *can be modulated non-competitively at an allosteric binding site.

### Conformation thesis

**Figure 11:**
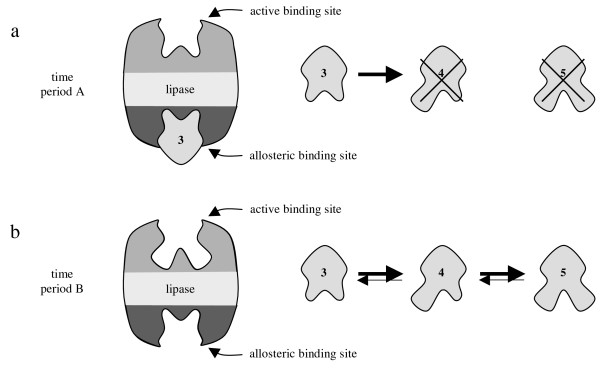
Conformation thesis of the allosteric modulation to explain the progress of the reaction using lipase from *M. miehei *(Fig. 9); a: during time period A compound **3 **binds allosterically modifying the active binding site.; b: during time period B the allosteric modulation is reversed.

The described allosteric modulation does not cause a complete inhibition of the lipase but a change in substrate selectivity. Allosterically bound diol **3 **modifies the active binding site of the lipase. As a result monoacetate **4 **cannot be acetylated during time period A. In spite of this inhibition the lipase acetylates diol **3 **without a change in activity. Thus, binding of diol **3 **at the allosteric binding site does neither result in blocking of the active site nor in complete inhibition of the enzyme as described in literature generally [15]. In fact the allosteric binding of diol **3 **leads to a modified conformation of the lipase, which allows only diol **3 **to be bound at the active binding site. Figure 11 shows the different conformations of the lipase during time period A (Fig. 11a) and during time period B (Fig. 11b) schematically.

These findings prove, that enzymes cannot just be switched on and off allosterically, but act as regulatory proteins controlling a metabolic system, a process that was so far only theorized [16]. The existence of a regulatory system controlled by a lipase, the conformation of which is changed allosterically, is described herein for the first time. It requires flexible parts in the tertiary structure of the enzyme, which were found in the computer simulated structure of the lipase from *M. miehei *[9].

**Figure 12:**
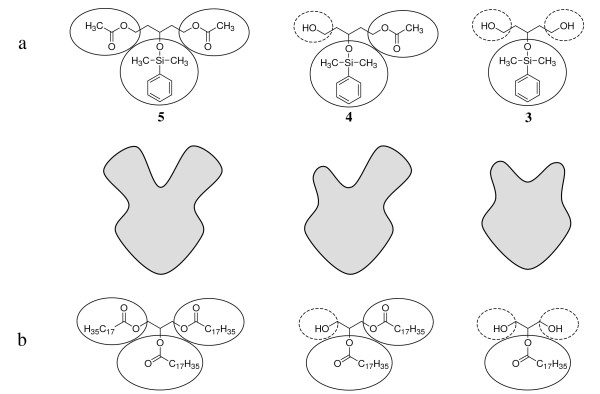
Structural analogy of the compounds used in the experiments (a) and natural substrates (b); solid lines encircle large lipophilic moieties (modified OH-groups), dashed lines encircle small hydrophilic moieties (free OH-groups); a: diacetate **5**, monoacetate **4 **and diol **3**; b: triglyceride TG, diglyceride DG and 2-monoglyceride 2-MG (stearic acid as fatty acid).

Since the lipase from *M. miehei *is well-investigated and used commercially [2], it is surprising that the allosteric modulation described herein was not detected so far. This is probably due to the fact that a number of parameters are necessary for the discovery. The reaction has to be carried out contrary to its natural direction and the progress has to be recorded by appropriate analytical techniques. In order to perform the acetylation with a necessary low lipase/inhibitor ratio the lipase needs to be highly active. A good choice is an immobilized lipase, since otherwise the reaction time is very long. Furthermore, we assume that the structures of the compounds used in experiments have to be very similar to the natural substrates (compare Fig. 12).

The allosteric binding site is an exciting new target for the development of ligands and even drugs, which bind reversibly or irreversibly. If the allosteric modulation can be stabilized permanently by use of such ligands, the resulting modified lipase will catalyze reactions with an increased selectivity. Even more challenging is the development of ligands that bind to the allosteric binding site without modifying the conformation of the lipase and thereby preventing other compounds from inducing the modulation too.

## Theory

### Biological function

The described discovery entails the question about the relevance of this regulation mechanism in nature. Natural substrates of lipases are triglycerides (TGs) that are hydrolyzed at their active site. The lipase from *M. miehei *belongs to the large class of sn-1,3 specific lipases [1,2], which catalyze the hydrolysis of ester groups in position 1 and 3 of glycerol preferentially. Thus, primary products are 2-monoglycerides (2-MGs) and free fatty acids (FFAs). 2-MGs are rearranged into 1-monoglycerides (1-MGs) by non-enzymatic acyl migration, which takes more time than the enzymatic process. Finally, the lipase catalyzes the hydrolysis of the resulting 1-MGs to give the end products glycerol and FFAs. Within mammalian cells the decomposition of 2-MGs is mainly carried out by monoacylglycerol lipase (MGL), which is expressed in excess. Cooperation of sn-1,3 specific lipases and sn-2 specific MGL ensures complete hydrolysis of TGs. Organisms use lipases in order to convert TGs into absorbable products. In contrast to TGs and DGs the produced MGs, glycerol and FFAs can be absorbed in the gastrointestinal tract. In this manner, upon hydrolysis by pancreatic lipase, 80% of consumed edible fat is absorbed as 2-MGs and 20% as glycerol along with the respective amounts of FFAs during human digestion [17-20]. It can be assumed that the lipase from *M. miehei *fulfills the same task of making TGs available as a source of energy.

A comparison of the natural substrates and their products with compounds **3**, **4 **and **5 **we used in the experiment leads to an apparent structural analogy. As described we investigated the reactivity of pentanetriol-derivatives instead of propanetriol (= glycerol)-derivatives. In this context diacetate **5 **corresponds to a triglyceride (TG), monoacetate **4 **to a diglyceride (DG) and diol **3 **to a 2-monoglyceride (2-MG) (Fig. 12). In contrast to 2-MGs, which are rearranged into 1-MGs by acyl migration, diol **3 **is stable due to its silylether-moiety.

**Figure 13:**
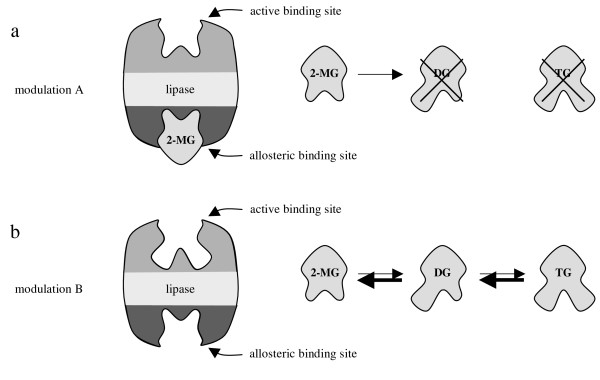
Conformation thesis of the allosteric modulation shown with natural substrates; TG = triglyceride; DG = diglyceride; 2-MG = 2-monoglyceride; a: In case of modulation A only 2-MGs can bind to the active site and can be transformed into DGs; b: In case of modulation B all compounds can bind to the active site. TGs and DGs can be hydrolyzed.

During evolution the lipase from *M. miehei *was certainly not developed to transform the synthetically produced compounds we used in our experiments. Since diol **3 **induces the allosteric modulation in the experiments, 2-MGs should accomplish the same task in nature (Fig. 13).

**Figure 14:**
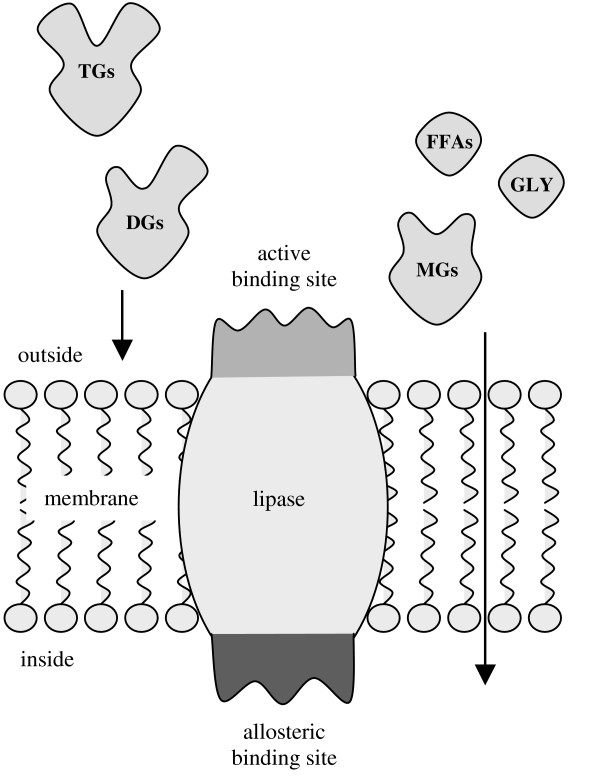
The allosteric regulation is exemplarily shown by means of a membrane bound lipase. Triglycerides (TGs) and diglycerides (DG) cannot pass through the membrane, whereas monoglycerides (MGs), free fatty acids (FFAs) and glycerol (GLY) which are the products of hydrolysis can be absorbed. Due to the allosteric mechanism the lipase hydrolyzes just as many TGs and DGs as are actually needed.

This assumption becomes even more striking considering the possible biological relevance of this regulation mechanism. 2-MGs are the primary products of lipase catalyzed hydrolysis and modify the lipase by allosteric modulation. Thereby they control the hydrolysis of TGs. This principle is generally known as feedback-inhibition for other enzymes. However the allosteric modulation as described herein does neither inhibit the lipase completely nor change its activity, but just prevent TGs and DGs from binding at the active site (Fig. 13a). The observation of this mechanism is only possible by forcing the reactions contrary to their naturally directed equilibria. Otherwise it cannot be distinguished from a loss in activity, since the modulator (2-MG) would be formed continuously resulting in a slowly increasing concentration and a slowly decreasing activity of the lipase. A model of the allosteric regulation mechanism is exemplarily shown in Figure 14 by means of a membrane-bound lipase [21,22].

In this model the active site of the lipase is at the cell surface whereas the allosteric binding site is located intracellularly. TGs and DGs are hydrolyzed extracellularly and the products (MGs, FFAs and glycerol) are absorbed. In this system the extent of extracellular hydrolysis is controlled by the concentration of the products inside of the cell. Thus, the lipase hydrolyzes just as many TGs and DGs as are actually needed. If the lipase from *M. miehei *is responsible for making fat available as a source of energy by catalyzing the conversion of glycerides, the allosteric modulation of the lipase enables the organism to control this procedure.

### Classification of lipases

As shown in with this article, obviously two different types of lipases are found in nature. Type-1 lipases, e.g. the lipase from *B. cepacia*, which hydrolyze TGs to a large extent without further control, and type-2 lipases, including the lipase from *M. miehei*, that regulate the conversion of glycerides depending on the concentration of substrates. The allosteric modulation can be more or less developed within different lipases, but for a better understanding only the two pure types are discussed in this article.

**Figure 15:**
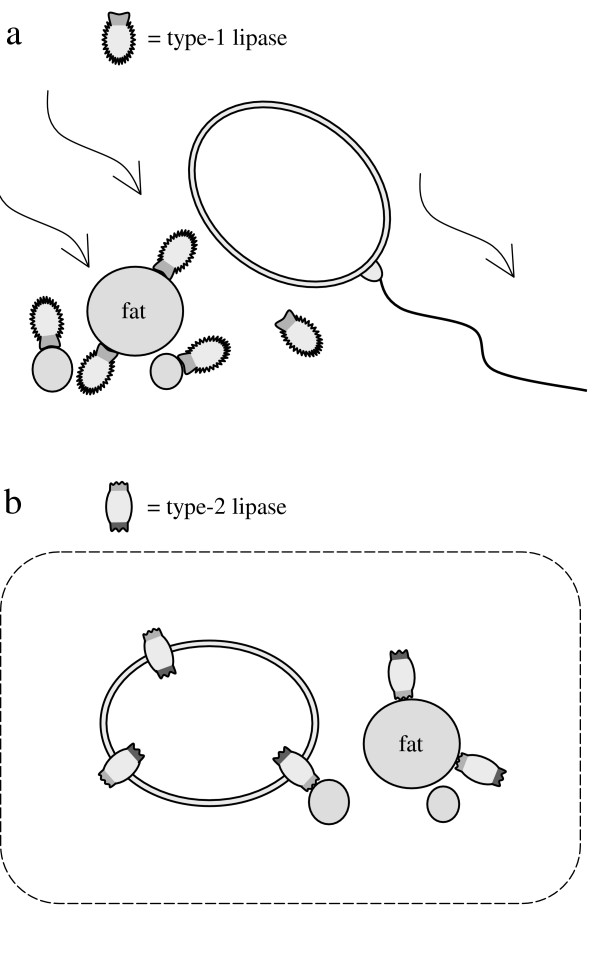
Organisms using type-1 or type-2 lipases to hydrolyze nutritional fat; a: Type-1 lipases hydrolyze fat to a large extent. The lipase secreting organism can depart by active or passive movement.; b: Type-2 lipases are regulated allosterically, therefore they hydrolyze fat depending on the concentration of products.

In order to avoid the detrimental effects of FFAs known as lipotoxicity [23,24] fatty acids (FAs) are bound to fatty acid binding proteins (FABPs) intracellularly. The same is done by albumin in the blood of mammals. However, this immobilization can be ensured only to a limited extent. Unlimited or uncontrolled hydrolysis of triglycerides would lead to concentration peaks of FAs, which become toxic as FFAs when FABPs are saturated. Therefore, a reason for the development of different types of lipases was possibly the need to avoid intoxication by FFAs released during hydrolysis of TGs. Their enantioselectivity is an indication for the possibility that the two types of lipases originate from phospholipases to make TGs available as a source of energy. All organisms that use lipases for this purpose must be able to control the extent of TG hydrolysis, whether the lipase is membrane-bound or secreted. The different mobility of organisms towards nutrient TGs should have stimulated the development of diverse lipases. An organism using unregulated type-1 lipases to hydrolyze TGs must be able to remove its absorbing membrane from the released products of hydrolysis when their amounts achieve toxic concentrations. Accordingly, these lipases are secreted and the organism departs from the released products by active movement or by drifting (Fig 15a). It is also possible that the nutriment containing the secreted lipase is carried along the absorbing membrane as it takes place in the intestinal tract of many organisms including humans [17-20].

In contrast to this an organism, which is immobile towards the products of TG hydrolysis can hydrolyze TGs and DGs profitably only by use of type-2 lipases (Fig 15b). The allosteric regulation mechanism of type-2 lipases keeps the concentration of the resulting products at a constant, non-toxic level irrespective of the amount of available TGs.

However both types of organisms shown schematically in Figure 15 have a major disadvantage in common. Unlike most of the organisms existing nowadays they cannot accumulate storage fat. This ability requires both hydrolysis and synthesis of TGs depending on demand and supply of nutriment. Storage fat can be accumulated either inside cells or inside inclusions surrounded by cells. In both cases only type-2 lipases can regulate the storage due to the immobility of the organism towards the products of TG hydrolysis. Even type-1 lipases secreted just on demand could not perform this task. Upon activation in order to hydrolyze storage fat a type-1 lipase could not be inactivated fast enough to stop this process as soon as nutritional fat becomes available again. As a consequence the concentration of FFAs would fluctuate reaching toxic peaks. Hence, all organisms using storage fat necessarily need allosterically regulated type-2 lipases and cannot exist without.

### Storage fat, lipases and endosymbiontic theory

**Figure 16:**
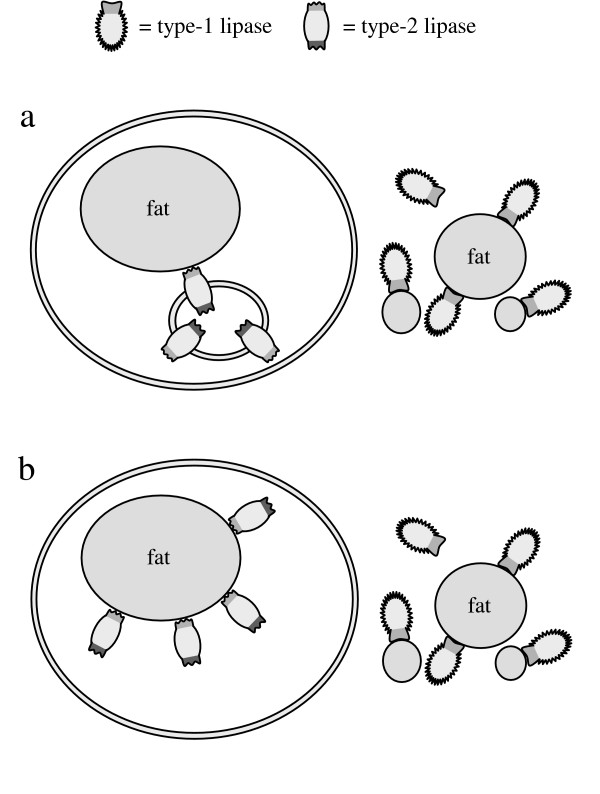
a: Supposed endosymbiosis of an organism containing type-2 lipases. As a result the allosteric regulation mechanism controls the concentration of products of hydrolysis and fat can be accumulated. This is a mutualistic symbiosis, as the effect is beneficial to both endo- and exosymbiont.; b: A new kind of organism results from genetic combination of the symbionts. The ability to accumulate and mobilize storage fat is maintained and provides a major benefit due to energy homeostasis.

Before discussing the control mechanism of fat metabolism concerning present-day organisms, the evolution of lipases that hydrolyze TGs intracellularly should be considered. Such lipases would soever hydrolyze phospholipids as well and thereby prevent the assembling of membranes. Additionally, both the development of a catalyst inside a cell, which does not contain any TGs to be hydrolyzed, and the accumulation of fat without a catalyst to re-hydrolyze it are hard to understand. An answer may be endosymbiosis, which is accepted as an essential event during evolution [25-27]. If an organism producing a type-2 lipase invades another cell, the type-2 lipase regulates the hydrolysis of TGs and keeps the concentration of products constant within the cell (Fig. 16a).

The arising new organism is able to accumulate TGs intracellularly during times supply of nutriment is plentiful and re-hydrolyze TGs on demand of energy. This is a mutualistic symbiosis, as the effect is beneficial to both endo- and exosymbiont. The endosymbiont is always provided with TGs and the exosymbiont can store TGs to use supply more efficiently. The new organism shown schematically in Figure 16 (a) has acquired an essential evolutional benefit by this metabolic symbiosis compared to those organisms shown in Figure 15. It is no longer dependent on continuous supply of nutritional fat and does not have to lapse into a resting state when food is missing. In fact, it is able to keep its metabolism running in times of reduced or ceased food supply not only surviving such time periods but even retaining the ability of reproduction. Constant energy supply is an essential requirement for the evolution of higher forms of life. The localization of type-2 lipases and their genes remain to be investigated, but are not relevant for further consideration. It can be presumed that the genetic information of the symbionts was possibly combined during evolution (Fig. 16b).

**Figure 17:**
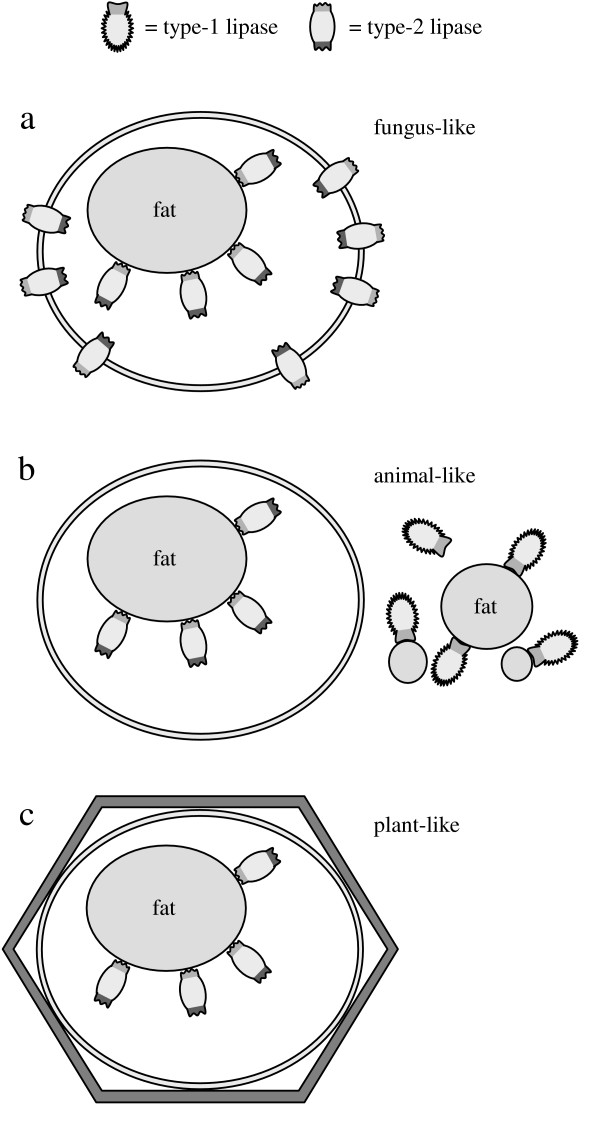
Possible formation of organisms upon endosymbiosis; a: An organism originating from symbiosis of an organism containing type-2 lipases and a cogenerous one is fungus-like; b: As the former exosymbiot used type-1 lipases to hydrolyze nutritional fat, an animal-like organism results.; c: If the animal-like organism does not need nutritional fat, since it can produce metabolic energy by photosynthesis, it can assemble a cell wall and becomes plant-like.

It is conceivable that the endosymbiotic process took place by invasion of a type-2 producing organism into a related one or into an organism producing type-1 lipases. In the former case a fungus-like (Fig. 17a) and in the latter case an animal-like organism is formed (Fig. 17b). If the animal-like organism is no longer dependent on ingestion due to the development of photosynthesis, type-1 lipases are not required any longer and the organism can assemble a cell wall whereby a plant-like organism results (Fig. 17c).

The regulation of fat metabolism in present-day fungi and plants is rather unexplored. The validity of the endosymbiontic process suggested herein remains to be investigated.

### Human fat metabolism

**Figure 18:**
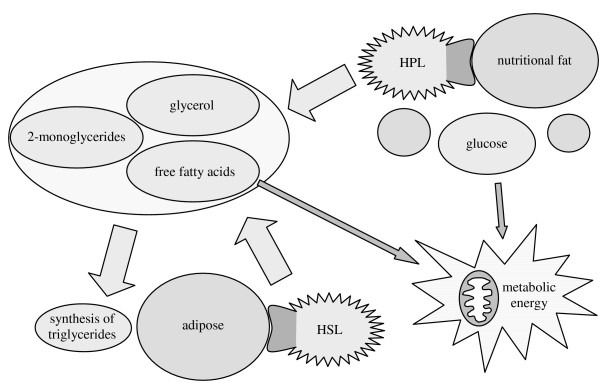
Schematic pathways of the human fat metabolism established until 2004. Nutritional fat is hydrolyzed by human pancreatic lipase (HPL) to produce 2-monoglycerides, glycerol and free fatty acids, which are absorbed. In addition to glucose these products provide metabolic energy directly or they can be transformed into adipose. Adipose is mobilized by hormone-sensitive lipase (HSL).

The scheme shown in Figure 17(b) is of particular relevance for the human fat metabolism. Humans should not only possess pancreatic lipase (HPL) as a type-1 lipase making nutritional fat available, but also a type-2 lipase that regulates the accumulation of adipose. Until recently, the hormone-sensitive lipase (HSL) was supposed to be the only lipase for hydrolysis of TGs [15]. The established TG metabolism is shown in Figure 18 schematically. HSL is activated by (nor)adrenaline induced phosphorylation and should be a type-1 lipase according to our classification.

Doubts about the assumption that HSL would be the only storage fat hydrolyzing lipase are entitled, since the release of adrenaline leads to several other effects in vivo, too. Furthermore, though activation by hormone induced phosphorylation is a kind of short-term regulation, deactivation of HSL can definitely not take place fast enough, since activation and deactivation requires a cascade of reactions [28,29]. As already described, this delay would temporarily lead to toxic concentrations of FFAs during ingestion. The obvious existence of another lipase than HSL for the mobilization of stored fat was shown by means of HSL-deficient mice [30-32]. These mice were viable and did not even develop overweight.

Recently Zimmermann et al. discovered a new TG lipase that was termed adipose triglyceride lipase (ATGL) (official name: patatin-like phospholipase domain containing protein-2, PNPLA 2) [33]. Currently it is under investigation that ATGL is identical to desnutrin [34] and phospholipase A2-ζ [35], which both were discovered in 2004, too. ATGL-deficient mice have an increased adipose mass and glucose use, accumulate large amounts of lipids, especially in the heart, and their energy homeostasis is highly defective, which proves this lipase to be crucial for the mobilization of adipose [36]. We assume that ATGL is the type-2 lipase we proposed to be responsible for the regulation of fat metabolism. ATGL contains a so-called patatin domain common to plant hydrolases, which is a further indication for our theory. Patatin is a protein from potato tuber (*Solanum tuberosum*) that is known to exhibit lipolytic activity [37]. The genetic relationship refers to the common endosymbiotical origin of these enzymes. For this reason type-2 lipases have to be more closely related to each other than to type-1 lipases, like HSL. Recently ATGL-like lipases were found in the fruit fly *Drosophila melanogaster *[38] and the yeast *Saccharomyces cerevisiae *as well [24]. A patatin-like lipase that initiates the breakdown of storage oil was discovered in seeds of the thale cress *Arabidopsis thaliana *[39]. All these lipases are closely related to the lipase from the mold *Mucor miehei *that was investigated in this article [39-41]. All of them are encoded by homologous genes and are responsible for energy homeostasis. Their pivotal role in lipolytic catabolism was shown in further studies [42].

According to our theory, these lipases must be type-2 lipases and are therefore regulated by allosteric modulation like the lipase from *M. miehei*. Another indication supporting our theory is the observation that patatin- and ATGL-like lipases paradoxically hydrolyze TGs and DGs only to a low extent in vitro [39,43]. This is explained by the allosteric mechanism presented in this article. Since the products of TG hydrolysis are not consumed in these experiments, the hydrolytic activity of the enzymes is inhibited to prevent toxic concentrations of FFAs.

**Figure 19:**
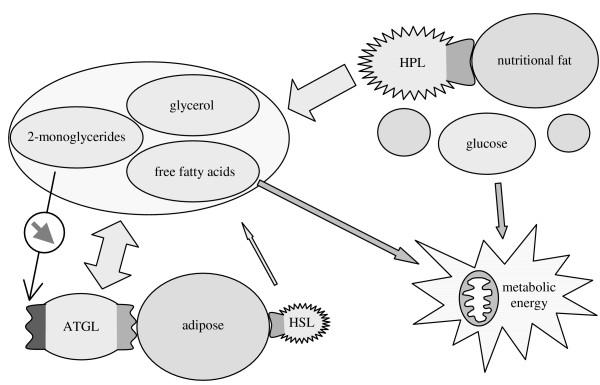
Scheme of the human fat metabolism as supposed according to our hypothesis. Human pancreatic lipase (HPL) hydrolyzes nutritional fat. The recently discovered adipose triglyceride lipase (ATGL) is rate limiting for the mobilization and storage of adipose. We assume that ATGL is regulated allosterically in the way we demonstrated herein. Irrespective of food supply this regulation mechanism keeps the concentration of the products of hydrolysis constant and maintains energy homeostasis. Hormone-sensitive lipase (HSL) is of minor importance.

We assume that the human fat metabolism is regulated by the same allosteric mechanism as demonstrated for the lipase from *M. miehei*. We further assume that the modulation is carried out by products of TG hydrolysis, which can pass membranes to provide cells with energy. Thereby nutritional fat, adipose and products of TG hydrolysis are balanced to ensure energy homeostasis. This homeostasis is shown schematically in Figure 19, which is an update of Figure 18 supplemented by the recently discovered ATGL.

The assumption of an allosteric ATGL regulation in the way we demonstrated herein leads to the following consequences. During the absence of food ATGL keeps the concentration of products of TG hydrolysis constant as generally described for type-2 lipases. When edible fat is ingested with nutrition, human pancreatic lipase (HPL) catalyzes its hydrolysis. The products of TG hydrolysis, in particular 2-MGs, immediately induce allosteric modulation of ATGL. This modulation leads to a modification of the active binding site preventing further hydrolysis of storage fat. The mechanism prevents fluctuations and thus toxic concentrations of FFAs [23,24]. Furthermore, ATGL is still able to store the temporary occurring excess of products of TG hydrolysis according to the 2-monoacylglycerol pathway, since the allosteric modulation does not inhibit the lipase completely but just prevents TGs and DGs from binding at its active site (compare Fig. 13). Thereby an excess of nutrition (energy) is stored as adipose and lipotoxicity is avoided. ATGL with the proposed properties of a type-2 lipase enables organisms, including humans, to maintain energy homeostasis irrespectively of food supply. The severe consequences of a dysfunction of this mechanism were shown with ATGL-deficient mice [36]. Due to the absence of a regulative lipase these mice are not provided sufficiently with energy when nutrition is interrupted even though their body fat is increased. As a result they show defective cold adaptation, which is life-threatening shortly after. The minor importance of HSL is shown with HSL-deficient mice as mentioned above. Obviously, it is just activated on demand, which is plausible due to its adrenaline induced activation associated to stress.

Another phenomenon is explained by our theory. Partial substitution of TGs by DGs in edible oils promotes weight loss and reduction of body fat [44]. These diacylglycerol enriched oils, which are termed DAG-oils [45], are commercially available as food additives. Due to acyl migration DAG-oils mainly consist of 1,3-disubstituted DGs, which means that the products of hydrolysis contain a lower concentration of 2-MGs. Since according to our hypothesis 2-MGs are a major factor for the allosteric modulation of ATGL, this process is reduced leading to an increased mobilization of adipose.

It is well established that glycerides are synthesized via the 2-monoacylglycerol pathway as well as the α-glycerophosphate pathway [46,47] and that the 2-monoacylglycerol pathway is preferentially used in adipose tissue [48,49]. 35 years ago it was shown that 2-MGs as well as 2-monoether analogues (2-MEs), which were used due to their resistance to hydrolysis, are acylated via the 2-monoacylglycerol pathway and seemingly inhibit the α-glycerophosphate pathway [50]. Because of the observed change of the pathway it was reasoned that 2-MGs may be important in the regulation of TG biosythesis, but the mechanism remained unknown. Today it is known that ATGL is expressed predominantly in adipose tissue [33] and the described findings can be explained with our theory. The used 2-MGs and 2-MEs induce modulation A of the lipase allosterically (compare Fig. 13a) and the only reaction that is catalyzed by the enzyme is the acylation of 2-MGs.

### The metabolic syndrome

The prevalently combined occurrence of obesity, hypertension, type 2 diabetes, high cholesterol level, gallbladder diseases, arteriosclerosis and cardiovascular diseases with an increased risk of heart attack and stroke is known as the metabolic syndrome [51-54]. Over the past decades the number of people suffering from the metabolic syndrome dramatically increased in industrialized countries. Therefore, numerous large-scale studies and statistics were performed and evaluated resulting in more than 12,000 articles and about 4,000 reviews on this subject during the last ten years (hits in PubMed). But in spite of intensive research activities reasons for the combined occurrence of these diseases as well as any correlations remain rather unknown. According to our hypothesis, a reduced expression rate of ATGL as a single reason is the crucial factor for the development of the various diseases within the metabolic syndrome

The activity of a certain amount of an enzyme is determined by short-term regulation, whereas the amount of an enzyme is controlled by the rate of gene expression, which is determined by long-term regulation [15]. The rate of expression depends on genetic disposition and age, but also on adaptation to the demand of metabolism. In case of low demand for mobilization of adipose, since energy is permanently available from nutrition, the expression rate of the corresponding lipase ATGL is accordingly decreased. Thus, deficient exercise and insufficient physical training combined with permanent nutrition result in down-regulation of ATGL. As the relative amount of 2-MGs produced by hydrolysis of TGs does not change, the available ATGL activity is decreased resulting in a slowdown of ATGL-catalyzed reactions. The most obvious consequences are an increased amount of adipose and the development of obesity.

If under the conditions of decreased ATGL activity the demand of metabolic energy is suddenly increased for a short time, the low amount of ATGL cannot provide the organism with sufficient energy. Due to Figure 19 there are some possibilities to circumvent the deficient energy state.

In order to improve the energy supply of the organism more blood must be transported into the consuming cells, since it contains a less amount of nutritive substances (FFAs). As a result the blood pressure is increased and hypertension is developed.

Nutritional fat can be used to a greater extent. For that purpose it has to be emulsified more efficiently by bile acids, which are derived from cholesterol. The increased demand of cholesterol results in an increased blood cholesterol level. Furthermore, the increased production of bile acids can cause gallbladder diseases.

HSL can be activated to a greater extent to hydrolyze a greater amount of adipose. As above-mentioned this leads to increased fluctuations of the concentration of FFAs. Toxic peaks result especially upon ingestion causing lipotoxicity. This effect is even reinforced, since in addition to that the ability of ATGL to store the excess of FFAs is reduced due to its lower amount. Since the solubility of FFAs in blood is limited, they are transported bound to albumin [15]. The temporarily increased concentration of FFAs in the blood increases the risk of interactions with the endothelial cells of the blood vessels. This mainly concerns the arteries due to the higher amount of FFAs therein. Deposits can be accumulated causing arteriosclerosis and an increased risk of heart attack and stroke. Stress combined with an increased concentration of adrenaline can also cause these effects, since HSL is activated to a greater extent.

The low energy level of the blood can also be compensated by increasing the concentration of glucose. A non-insulin-dependent hyperglycemia emerges which can be medicated but not cured (type 2 diabetes). Nutrition containing large amounts of carbohydrates or sugar also promotes the use of glucose instead of fat as a supply of energy, which results in the effects mentioned herein.

The suggestion to enhance physical exercise for prevention of the metabolic syndrome remains unchanged [55,56]. Maybe similar effects can be achieved by a change of the eating habits, which often include an all-day long permanent nutrition. Periods of hunger also result in an increased stimulation of ATGL expression by the need to immobilize adipose.

According to our theory a medicinal treatment of the metabolic syndrome seems to be difficult, since the ATGL expression rate cannot be increased in this way. Nevertheless, two possibilities should be mentioned here. The concentration of 2-MGs resulting from the hydrolysis of nutritional fat could be decreased by use of a 2-MG selective lipase, like the lipase from *Bacillus stearothermophilus *[57,58], which has to be applied in a gastro-resistant form. Secondly, ligands could be developed that bind to the allosteric binding site without modifying the conformation of the lipase and thereby preventing other compounds from inducing the modulation.

In conclusion, the diseases within the metabolic syndrome are described in a highly simplified manner. However, explanation of the metabolic syndrome by just one reason, a decreased ATGL expression, represents a striking idea. Although, a lot of work has to be done to prove or disprove this theory and to find and optimize potent ATGL ligands binding allosterically, this theory should stimulate further research in the field of fat metabolism, which is a very up to date topic [59]. Moreover, this theory provides a novel starting point for the development of innovative therapeutic strategies [60].

## Methods

### Chemical compounds

Dimethyl 3-hydroxyglutarate **1 **(CAS 7250-55-7) was silylated and the resulting dimethyl 3-(dimethylphenylsilyloxy)glutarate **2 **was reduced by LiBH_4 _to give 3-(dimethylphenylsilyloxy)pentane-1,5-diol **3 **as described in literature [13]. Enzymatically produced [3-(dimethylphenylsilyloxy)-5-hydroxypentan-1-yl] acetate **4 **and [3-(dimethylphenylsilyloxy)pentane-1,5-diyl] diacetate **5 **are also characterized in this article.

### Reaction courses

The following procedures were carried out to observe the progress of the reactions according to literature [13].

#### Enzymatic transformations

Employed lipase preparations:

Amano PS-CII (lipase from *Burkholderia cepacia*) by Aldrich^®^

Lipozyme^® ^(lipase from *Mucor miehei*) by Fluka^®^

General:

In a 50 mL two-necked flask diol **3 **(about 280 mg) was dissolved in TBME (30 mL) and the respective lipase was added (0.50 or 1.00 weight equivalents). To avoid damage of the ceramic particles the reaction mixture was stirred with a KPG-stirrer (100 rpm). The given reaction temperature was adjusted by a cryostat (20°C, -10°C or -40°C) and the reaction was started by addition of the respective amount of isopropenyl acetate (IPA) (5.00 or 15.0 equivalents). The exact reaction conditions are listed in Table 1.

**Table 1:** Reaction conditions of the enzymatic transformations

Figure	lipase preparation	amount of lipase [mg]	amount of diol **3 **[mg]	amount of IPA [μl]	temperature [°C]
5 a, b	Amano PS-CII	139.5	288.7	629	20
5 c, d	Lipozyme^®^	138.0	275.9	597	20
6 a, b	Amano PS-CII	291.8	291.5	1715	-40
6 c, d	Lipozyme^®^	142.0	283.5	614	-10

#### Preparation of samples

In order to analyze the transformation samples (100 μL) were taken from the reaction mixture, filtered through a membrane filter (0.45 μm) and the solvent was evaporated in a nitrogen stream during 2 min. The residue was dissolved in 100 μL of acetonitrile for HPLC method 1 and in 100 μL of a n-hexane/propan-2-ol 9:1 mixture for HPLC method 2.

### HPLC Methods

#### Method 1 (achiral)

column: Merck LiChrospher 100 RP-18e (5 μm); 125 - 4 mm.

mobile phase: acetonitrile/water 50:50; 1 mL/min.

detection: λ = 264 nm for 16 min.

retention times (rt): rt (**3**) = 1.9 min; rt (**4**) = 4.1 min; rt (**5**) = 12.4 min.

scaling factors (sf): sf (**3**) = sf (**4**) = sf (**5**) = 1.00 [n (%)/area (%)].

#### Method 2 (chiral)

column: Daicel Chiralpak AD-H (5 μm); 250 - 4.6 mm.

mobile phase: n-hexane/propan-2-ol 51:1; 1 mL/min.

(After 40 min the column was purged with n-hexane/propan-2-ol 9:1 and re-equilibrated.)

detection: λ = 264 nm for 40 min.

retention times (rt): rt ((*S*)-**4**) = 25.0 min; rt ((*R*)**-4**) = 27.5 min.

The rt can be increased by using n-hexane/propan-2-ol 54:1 to rt ((*S*)-**4**) = 27.5 min; rt ((*R*)**-4**) = 32.5 min.

scaling factors (sf): sf ((*S*)-**4**) = sf ((*R*)-**4**) = 1.000 [n (%)/area (%)].

### Computer simulations

The Microsoft^® ^Excel table used to simulate competitive desymmetrizations (Fig. 5e, f, Fig. 6e, f and Fig. 8a, b, c, d) was published recently and is available as supplementary material [14]. The abbreviations of compounds **E**, **P **and **W **used in that article simply have to be replaced by the compound numbers used herein as follows: **E **= **3**; **P**_**S **_= (*S*)-**4**; **P**_**R **_= (*R*)-**4**; **W **= **5**.

In order to simulate a non-competitive reaction (Fig. 8e, f) the computation has to be modified. Differing from literature [14] the changes of the amounts of substances during a time interval are calculated as follows: Δn(**3**)_i _= - *a *; Δn((*S*)-**4**)_i _= *a*·*d *; Δn((*R*)-**4**)_i _= *a*·*e *; Δn(**5**)_i _= 0.

## Competing interests

In order to protect the procedure of preventing lipases from allosteric modulation the authors applied for a German national patent (DE102005029115A1).
